# Genus *Viburnum*: Therapeutic Potentialities and Agro-Food-Pharma Applications

**DOI:** 10.1155/2021/3095514

**Published:** 2021-07-16

**Authors:** Javad Sharifi-Rad, Cristina Quispe, Cristian Valdés Vergara, Dusanka Kitic, Milica Kostic, Lorene Armstrong, Zabta Khan Shinwari, Ali Talha Khalil, Milka Brdar-Jokanović, Branka Ljevnaić-Mašić, Elena M. Varoni, Marcello Iriti, Gerardo Leyva-Gómez, Jesús Herrera-Bravo, Luis A. Salazar, William C. Cho

**Affiliations:** ^1^Phytochemistry Research Center, Shahid Beheshti University of Medical Sciences, Tehran, Iran; ^2^Facultad de Ciencias de la Salud, Universidad Arturo Prat, Avda. Arturo Prat 2120, Iquique 1110939, Chile; ^3^Centro de Investigación de Estudios Avanzados del Maule, Vicerrectoría de Investigación y Postgrado, Universidad Católica del Maule, Chile; ^4^Faculty of Medicine, Department of Pharmacy, University of Niš, Ave. Zorana Djindjica 81, 18000 Nis, Serbia; ^5^Departament of Pharmaceutical Sciences, State University of Ponta Grossa, 84030900, Ponta Grossa, Paraná, Brazil; ^6^Department of Plant Sciences, Quaid-i-Azam University, Islamabad, Pakistan; ^7^Pakistan Academy of Sciences, Islamabad, Pakistan; ^8^Department of Pathology, Lady Reading Hospital Medical Teaching Institution, Peshawar (25000), KP, Pakistan; ^9^Institute of Field and Vegetable Crops, National Institute of the Republic of Serbia, Alternative Crops and Organic Production Department, Maksima Gorkog 30, 21000 Novi Sad, Serbia; ^10^Faculty of Agriculture, Department of Field and Vegetable Crops, University of Novi Sad, Trg Dositeja Obradovića 8, 21000 Novi Sad, Serbia; ^11^Dipartimento di Scienze Biomediche, Chirurgiche ed Odontoiatriche, Università Degli Studi di Milano, Via Beldiletto 1, 20142 Milan, Italy; ^12^Dipartimento di Scienze Agrarie e Ambientali, Via Celoria 2, 20133 Milan, Italy; ^13^Departamento de Farmacia, Facultad de Química, Universidad Nacional Autónoma de México, Ciudad Universitaria, Ciudad de México 04510, Mexico; ^14^Departamento de Ciencias Básicas, Facultad de Ciencias, Universidad Santo Tomas, Chile; ^15^Center of Molecular Biology and Pharmacogenetics, Scientific and Technological Bioresource Nucleus, Universidad de La Frontera, Temuco 4811230, Chile; ^16^Department of Clinical Oncology, Queen Elizabeth Hospital, Kowloon, Hong Kong

## Abstract

The genus *Viburnum* (Adoxaceae, Dipsacales) is of scientific interest due to the chemical components and diverse biological activities found across species of the genus, which includes more than 230 species of evergreen, semievergreen, or deciduous shrubs and small trees. Although frequently used as an ornament, the *Viburnum* species show biological properties with health-promoting effects. Fruits, flowers, and barks of certain species are used for pharmaceutical purposes or as cooking ingredients, hence containing biochemical compounds with health-promoting activity such are carotenoids, polyphenols, and flavonoids. However, its taxonomical determination is difficult, due to its wide distribution and frequent hybridizations; therefore, an objective classification would allow us to understand its biological activity based on its phytochemical components. More than sixty phytochemical compounds have been reported, where vibsanin-type diterpenes and their derivatives are the most prevalent. Leaves and twigs of *V. dilatatum* contain the largest number of phytochemicals among the genus. Through preclinical evidence, this study provides insight regarding antioxidant, antibacterial, anti-inflammatory, cytotoxic, and anticancer activities of genus *Viburnum*.

## 1. Introduction

The genus *Viburnum* (Adoxaceae, Dipsacales) is comprised of more than 230 species of evergreen, semievergreen, or deciduous shrubs and small trees distributed primarily within the temperate forest regions of the northern hemisphere but also in the mountains of Central and South America, Southeast Asia (Philippines, Malaysia), and southeastern Australia and Tasmania.

Although the species of the genus are generally well adapted to mesic forest environments, particular species have been reported to inhabit both, cold boreal forests, and tropical rainforests, as is the case of *Viburnum edule* and *Viburnum amplificatum,* respectively. The regions of highest species diversity are eastern Asia and Latin America [1–4]. *Viburnum* was established by Linnaeus, classified in the Caprifoliaceae family [5] and soon after reclassified in its own family Viburnaceae [6]. Based on the Dipsacales phylogeny research, *Viburnum* was subsequently listed in Adoxaceae, together with *Adoxa*, *Sinoadoxa*, *Tetradoxa*, and *Sambucus* [7] This classification is widely accepted today [1, 4, 8]. Nevertheless, certain plant classification systems, e.g., the one from Takhtajan [9], still recognize *Viburnum* as a member of the Viburnaceae family.

Leaves of the *Viburnum* species are simple, petiolate, and opposite and rarely whorled. The small hermaphroditic flowers form paniculate or umbellate inflorescences of 15-500 flowers each. Marginal flowers are sometimes sterile, and their purpose is to attract pollinators. The plants are self-incompatible. The androecium consists of five stamens. Filament bases are attached to corolla petals. The gynoecium is of three carpels. Two of the three inferior ovaries are aborted. The fruit is a red to purple black single-seeded drupe. Floral nectaries located at the gynoecium apex additionally differentiate viburnums from another Adoxaceae [10–14].

Taxonomical determination of the genus *Viburnum* is difficult, due to its wide distribution and frequent hybridizations, both natural and horticultural [15, 16]. Traditionally accepted classification is based on plant morphology (inflorescences, flowers, extrafloral nectaries, trichomes, and pollen grain exine) and recognizes the sections *Solenotinus*, *Viburnum*, *Pseudotinus*, *Tomentosa*, *Tinus*, *Megalotinus*, *Lentago*, *Oreinotinus*, *Odontotinus*, and *Opulus Opulus* ([17, 18]); however, several mostly regional-oriented section level revisions were made ([3] and references therein). Besides, plant morphology, taxonomical values of the phytochemical amentoflavone isolated from *Viburnum* leaves and branchlets [19], as well as anatomical characteristics of fruits, cork, assimilating parenchyma, and leaf epidermal cells [20, 21], were also assessed. More recently, assessments of *Viburnum* diversification have been made at a molecular level [3, 4, 8, 22, 23]. Clement et al. [24] proposed a *Viburnum* phylogenetic classification and provided formal phylogenetic definitions for 30 clades.

Many viburnums are of high ornamental value. Fruits, flowers, and barks of certain species are used for pharmaceutical purposes or as cooking ingredients, hence containing biochemical compounds with health-promoting activity such as carotenoids, polyphenols, and flavonoids. Many viburnums are of high ornamental value. Fruits, flowers, and barks of certain species are used for pharmaceutical purposes or as cooking ingredients, hence containing biochemical compounds with health-promoting activity such are carotenoids, polyphenols, and flavonoids. These biochemical components are responsible for the main biological activities of Viburnum plants, and although the antioxidant, antibacterial, anti-inflammatory, and cytotoxic activity have been well documented, some current research also links to the chemical components found in Viburnum exerting protection and treatment against diseases. Chronic diseases including diabetes [25], cancer [26], Alzheimer's [27], and all diseases that can be caused by a clot [28].

Substantial research has been carried out in relation to the mechanisms responsible for the synthesis, location, and accumulation of bioactive compounds. The most widely researched species are *V*. *opulus*, *tinus*, *lantana*, and *orientale*; although, various biochemical constituents have also been isolated from *V*. *arboricolum*, *awabuki*, *ayavacense*, *betulifolium*, *cylindricum*, *davidii*, *dilatatum*, *erosum*, *furcatum*, *grandifolium*, *japonicum*, *jucundum*, *luzonicum*, *odoratissimum*, *phlebotrichum*, *pichinchense*, *prunifolium*, *rhytidophyllum*, *suspensum*, *urceolatum*, and *wrightii* [21, 29–31]. Studies undertaken to assess within-species variability in terms of bioactive compounds profile and content [32–34] facilitate breeding and subsequent growing of viburnums as pharmaceutical crops.

## 2. Traditional Uses

Plants and humans are in a forever codependent relationship. Plants are considered the lungs of the earth but also provide food, shelter, timber, medicines, etc. for humans. Medicinal plants represent a rich reservoir of bioactive chemicals of therapeutic potential [35]. A review of the scientific literature reveals multiple applications from medicinal plants of the genus *Viburnum* (Table 1), implying the need for further research and documentation.

## 3. Phytoconstituents

Although *Viburnum* initially diversified in East Asia, other regions such as eastern North America, the mountainous regions of Mexico, Central America, and northern South America are also viewed as centers of high diversity. Most of the American *Viburnum* species seem to have derived from Asian clades, while the species in Latin America appear to be directly related to those in the eastern United States [59]. In this way, the genus represents a classic pattern of disjunct distribution between the Old and the New World.

Roughly 200 species have been reported for the genus *Viburnum*, among shrubs and trees, distributed mainly on the Asian side, used primarily in traditional medicine for the treatment of diseases such as rheumatoid arthritis, cough, diarrhea, tumefaction, swelling, kidney cramps [60], antitumor, antimicrobial, antioxidant, antihyperglycemic, anti-inflammatory, and neuroprotective activities [61]. Leaves, flowers, and fruits are currently being used in Turkish folk medicine [62].

The study of *Viburnum* is of scientific interest due to the chemical components and diverse biological activities found across species of the genus. Although the highest number of chemical compounds has been found in leaves, the presence of phytochemicals has also been reported in fruits, roots, and seeds (Table 2). *V*. *awabuki*, *V*. *dilatatum*, *V*. *fordiae Hance*, *V*. *odoratissimum*, and *V. opulus* have the highest amount of extracted phytochemical constituents (diterpenes, triterpenes, iridioides, monoterpenes, sesquiterpenes, flavonoids, lignans, etc.) and are dependent on the specific part of the plant from which the extract is obtained, as well as the type of solvent used.

**Figure 1:**
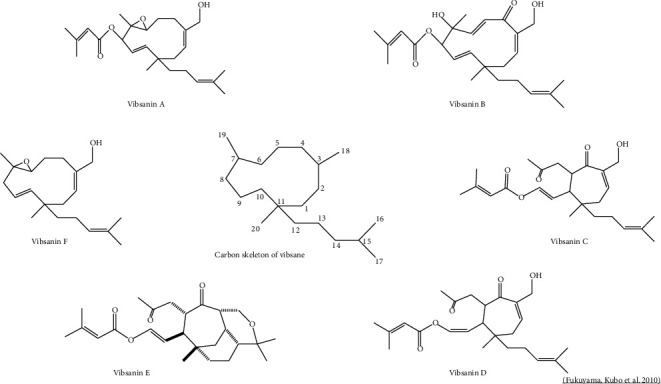
Chemical structure of vibsane-type diterpenoids components reported on the genus *Viburnum.*

In the case of *V*. *awabuki*, few studies have been found regarding the description of its phytochemical constituents (Table 2). The number of chemical compounds reported amounts to more than sixty, where vibsanin-type diterpenes and their derivatives are the most prevalent (Figure 1). These phytochemicals can also be present in species such as *V*. *odoratissimum* and *V*. *suspensum*. These diterpenoids can chemically be eleven-membered ring, seven-membered ring, and rearranged types, represented by vibsanine B, vibsanine C, and neovibsanine A, respectively. Some vibsane-type diterpenoids have exhibited increasing biological activities, and their challenging structures combined with attractive neurotrophs have drawn synthetic attention [29]. Table 2 reports the compounds found in *V*. *awabuki* corresponding to methanolic extracts of leaves and twigs of the plant [29, 31]. Other compounds such as triterpenoid derivatives, sesquiterpenes (such as awabukinol), specific flavonoids of the catechin type, coumarin derivatives, and lignans have also been observed.

Leaves and twigs of *V. dilatatum* contain the largest number of phytochemical constituents within the genus (Table 2). The triterpenoids viburnols (Viburnol A, B, C, D, E, F, G, H, I, J, and K), viburnudienone, and viburnenone are present in leaves, as well as, flavonoids, phenolic, and lactone type compounds [31]. The main compounds in the essential oils of *V*. *dilatatum* flowers are phenethyl alcohol, 3Z-hexenol, and l-linalool [71]. Glycosylated phenolic compounds of the jiamizioside type (A, B, C, and D) and anthocyanidins and quercetin flavonoids have also been found in methanolic and squeezed juice extracts of fruits. Compounds derived from phenylpropanoids such as 5-O-caffeoyl-4-methoxyl quinic acid and polyphenolic esters (chlorogenic acid) were also reported in the fruits, along with cyanidin 3-sambubioside, 5-caffeoyl quinic acid [3, 69, 70], cyanidin 3-glucoside, 4-methoxy chlorogenic acid, chlorogenic acid, and quercetin [3, 70]. These extracts show a significant antioxidant activity related to the described compounds [113]. Only one study reports the presence of the compound, 2-(-glucopyranosyloxy)-benzyl 3-(-glucopyranosyloxy)-benzoate, in the methanolic extract of roots [72].

**Figure 2:**
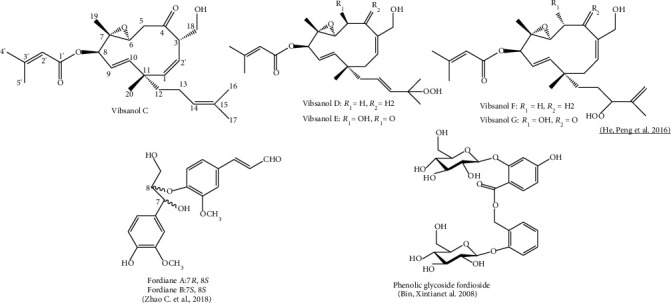
Chemical structure of the visanin components reported on the genus *Viburnum.*

This field of research is relatively novel and phytochemicals in *V*. *fordiae* have been found in leaves, branches, and fruits (Table 2) [61, 79]. First reports of the phytochemical compounds in *V*. *fordiae* were made in methanolic extracts of leaves. Compounds such us glycosylated phenolic type (fordioside), lignan glucoside (alangilignoside D), alcoholic *β*-glucoside (salicin), sand tilbenoid (rhapontigenin) were reported [67]. Recent studies describe essential oils [114], terpenoids, neolignans [81], and 52 phenolics [67] in stems, leaves, and roots. These compounds have shown to exhibit weak or moderate antioxidant, anti-inflammatory, and *α*-glucosidase inhibition properties [78, 81]. Neolignan glycosides, viburfordosides A–I, neolignans, fordianes A and B (Figure 2), and analogues present in the ethanolic fruit extract have been described to serve as functional foods and for the prevention and treatment of type 2 diabetes (T2D) [81].

Studies have described a wide range of phytochemical constituents for members of the genus *Viburnum*, such as diterpene, triterpene, and flavonoid type for *V*. *odoratissimum*. vibsanin, vibsane [89], and vibsanol, and their derivates present in leaves (Figure 2) [88, 90, 93, 94], twigs [91], and branches [92], have been found using methanol and ethanol as primary extraction solvents (Table 2). Triterpenes 6*β*-hydroxy-3-oxolup-20(29)-ene-27, 28-dioic acid, and 6*α*-hydroxy-3-oxolup-20(29)-ene-27, 28-dioic acid [31, 94] have been reported in leaves. Volatiles in *V*. *odoratissimum* flowers consist of esters, alkanes, ketones, alcohols, aldehydes, and acids. The main compounds in flowers were methyl o-anisate, heneicosane, methyl salicylate, 1-[2-(1-hydroxy-1-methylethyl) cyclopropyl] ethanone, linalool, nonanal, and methyl palmitate [95]. Compounds such as triterpenes and trans-phytol fatty alcohol have been detected in the root [96].

Studies carried out on fruit juice [100, 103, 104], aqueous methanolic extracts [98], acidic mixtures of water/methanol [102], and mixtures of methanol/acetone/water [99] have been assessed in *V*. *opulus* (Table 2). It is one of the few species where the volatile compounds of its fruits are described [115]. Mass spectrometry analysis has identified nine components in *V. opulus* juice, using ultra high-performance liquid chromatography (UPLC) coupled to quadruple time-of-flight mass spectrometers (QTOF-MS) [100]. *Viburnum* fruits have been reported to contain lipids, pectins, proteins, lipid compounds (carotenoids, essential oils, steroids, and saponins), tannins, flavonoids, and anthocyanins-type polyphenols. *V*. *opulus* fruits have a higher content of carotenoids, polyphenols, flavonoids, steroids, and pectins than *V*. *lantana*; the latter species being characterized by a higher content of proteins, saponins, and essential oils. Metabolites in *V*. *opulus* fruits have been found in different layers of the pericarp with greater amounts in the skin [21]. Phenolic compounds have been reported in fruit juice via hydrochloric acid analysis [101] while triterpenic compounds have been obtained from seeds after triterpene esters hydrolysis (terpenes of the sterols-I type, triterpenyl alcohols and their derivatives-II) [105]. In a review published in 2010, it was reported that only 3-O-caffeoylquinic acid and 5-O-caffeoylquinic acid were isolated from the *V*. *opulus* [31]. The presence of phenolic compounds, anthocyanins, and others constituents (Table 2) have been reported in leaves and hydrodistillation extracts of dried *V*. *opulus* plants [83, 97].

In this context, the genus *Viburnum* contains chemical compounds grouped in diterpenes, triterpenes, iridoids, monoterpenes, sesquiterhytopenes, flavonoids, lignans, phenols, coumarins, lactones, and alkaloids. Among the chemical contents of *Viburnum*, vibsane-type diterpenoids are characteristic of the genus, as have not been found in other higher plants. Compounds of the type vibsanin A-F, vibsanol (C-F), phenolic glycoside fordioside, fordiane A and B, and their derivatives have also been highlighted (Figure 2). The base structure of the visanin (A-F) compound group corresponds to a vibsane carbon skeleton, with a 20-carbon structure (Figure 2).

Another key aspect is the type of solvent used for the phytochemical extraction, where water, methanol, ethanol, and their mixture are used in higher proportion. In some cases, less polar solvents such as ethyl acetate or n-butanol are used, from branches and leaves. Chloroform has been used in leaves, while benzene or petroleum ether for the extraction of phytochemicals from branches.

Table 2 shows the main phytochemicals found in the genus *Viburnum*, as well as the type of extract obtained, the part of the plant used and the biological activity.

## 4. Biological Activities

### 4.1. Antioxidant Activities of *Viburnum* Plants (*In Vitro* Studies/*In Vivo* Studies)

Many traditionally used medicinal herbs exert a beneficial impact on human health thanks to their antioxidant potential. Phenolic compounds, commonly found in plants, are the largest group of natural antioxidants. Plants produce them to protect their cells from oxidative damage caused by oxygen radicals and molecular excitation [116, 117]. *Viburnum* plant species have been extensively studied *in vitro* and *in vivo* assays. Most antioxidant studies relate to *Viburnum* fruits and juices, being *V*. *opulus* the most investigated plant species. According to the numerous published results, it can be said that *Viburnum* species and their products are exceptional antioxidants finding their place as naturally safe agents.

#### 4.1.1. *In Vitro* Studies

The fruit juice of *V. opulus* (from the Eastern Black Sea Region, Turkey) had a prominent activity in the 2,2′-azino-bis (3-ethylbenzothiazoline-6-sulfonic acid) (ABTS) and ferric reducing antioxidant power (FRAP) system in comparison to methanol, acetonitrile, and aqueous extracts of fruits skin and seeds, where the seed extract contained a higher number and quantity of antioxidant compounds. Coumaroyl-quinic acid, chlorogenic acid, procyanidin B2, and procyanidin trimer were dominant in the juice [100]. Turkish *V. opulus* fruit, flesh, and seeds were analysed by the 2,2-diphenyl-1-picrylhydrazyl (DPPH) method. Fruits and seeds expressed a more potent effect with EC_50_ of 2.35 mg/mg DPPH, as compared to EC_50_ of 24.56 mg/mg DPPH found in the flesh [103]. Antiradical activity tests in DPPH and ABTS, and antilipoperoxidation in the *β*-carotene/linoleic acid bleaching, were applied in aqueous and methanol extracts of the same species. Methanol extract of dried fruits had a greater performance in the DPPH test with IC_50_ of 0.104 mg/ml, while that of fresh fruits scavenged more ABTS radicals. All extracts inhibited the linoleic acid peroxidation, thus protecting the loss of *β*-carotene. The methanol extract reduced the ferric (III) to the ferro (II) form more effectively than the aqueous extract [118]. Sagdic et al. [119] also tested the fruits' methanol extract of Turkish *V*. *opulus* using the phosphomolybdenum complex method and found a value of 315.50 ± 8.2 mg/g in relation to the ascorbic acid. The antioxidant capacities of aqueous extracts of commercially available fruits, bark, and flowers of *V*. *opulus* from Poland were assessed by ABTS, hydroxyl radical scavenging, and peroxyl radical scavenging (ORAC) and FRAP techniques. The bark aqueous extract displayed the highest antioxidant capacity, followed by flowers and fruits. Strong correlations were found between total phenolic, flavanol, and proanthocyanidin contents with these assays [34]. The same authors investigated the antioxidant capacities of *V. opulus* fruits, flowers, and bark ethanol extracts by measuring of ABTS-, hydroxyl- (HORS), peroxyl- (ORAC), and superoxide- (SORS) free radicals scavenging and reducing power (FRAP). The antioxidant activity of different parts of the herb was in the following order for HORS, SORS, and ORAC tests: bark>flowers>fruits, and for ABTS and FRAP tests: bark>fruits>flowers. The dominant compound in *V. opulus* bark was (+)-catechin, while chlorogenic acid was dominant in flowers and fruits [34].

Andreeva et al. [120] determined the antioxidant potential of Russian *V*. *opulus* bark extracts using the cathode voltammetry method measuring the relative decrease in the oxygen electroreduction. Results showed that the ethyl acetate fraction of the 70% ethanol extract expressed a higher antioxidant potential than chloroform and aqueous fractions, 70% and 30% ethanol extract, respectively. Bubulica et al. [121] conducted an antioxidant effects screening across several extracts of *in vitro* assays of Romanian plants. The authors found that the *V. opulus* methanol bark extract produced an antiradical effect against DPPH radicals with IC_50_ of 0.918 ± 0.46 mg/ml, as well as ferrous ion chelating effects with IC_50_ of 1.865 ± 0.05 mg/ml. Additionally, the antioxidant activity of the ethanol extract of the fruits was determined by the ABTS test. After 24 h of refrigerating, a 16% decrease in the antioxidant effect was recorded, followed by a 22% increase in the next 24 h, showing no correlation between the total the antioxidant potential and the phenol content in the extract during storage [122]. Paşayeva et al. [123] suggests that Turkish *V. opulus* fruits could be used as a neuroprotective agent. Antioxidant properties of both the decoction ethanol extract and fruit juice were used against hydrogen peroxide-induced oxidative stress in human SH-SY5Y neuronal cells. The Polish *V. opulus* fresh juice and extracts acted as scavenging potential agents toward ABTS and peroxyl radical cations estimated by ABTS and ORAC assays, expressed using Trolox equivalents (TE). The phenolic-rich fraction from fresh juice was compared to the methanol-acetone extract from pomace. The former was the most active in all identified phenol compounds (flavanols, falvonols, hydroxycinnamic acids, and anthocyanins) with values within the range of 2619.59 ± 123.1 and 7810.29 ± 342.3 *μ*g TE/g. Also, the *V. opulus* extracts had powerful chemopreventive effects against oxidative stress in Caco-2 cells induced by tert-butylhydroperoxide and against DNA damage through the repair induction after cell exposure to hydrogen peroxide and methylnitronitrosoguanidine [99].

Studies have compared the antioxidant effects of different *V*. *opulus* genotypes and cultivars. Kraujalytė et al. [101] reported that *V*. *opulus* var. *sargentii* had the highest radical scavenging capacity (77.5%), followed by *V*. *opulus* var. *americanum*, and *V*. *opulus* P3. The strongest activity in a FRAP test system was observed in *V*. *opulus* var. *sargentii* juice while the weakest was expressed by the ‘Shukshinskaya' cultivar. The *V*. *opulus* var. *sargentii* juice was most effective in the ORAC method with the highest TEAC (Trolox equivalent antioxidant capacity) value, while ‘Shukshinskaya' was powerless as observed in the previous test. Moskalets et al. [124] assessed the antioxidant capacity of Ukrainian *V*. *opulus* fruit varieties using a Blisar A analyser. The antioxidant activity was expressed through gallic acid in a range between 387 and 540 mg%. The cultivar with the maximal tested effect was ЕF 3-10-2010. Furthermore, approximately 10 genotypes of Turkish *V*. *opulus* fruits showed antioxidant potential, marked SIV 1-10 by a FRAP test system. SIV genotypes in the forms of acetone extracts displayed effects in a narrow range from 21.02 ± 2.6 to 34.90 ± 4.5 *μ*mol TE/g, where SIV-10 had the greatest capacity, suggesting the higher synthesis or accumulations of phenolics and others antioxidant compounds in fruits [33]. Scavenging effects of fruit porridge (25%) of *V*. *opulus* var. *edule* cultivars Leningradskaya otbornaya, Souzga, and Taezny rubiny on nitric oxide, superoxide anion, hydroxyl radical, and lipid peroxidation inhibition, were moderate from 11.20 to 28.50%. Fruits' total antioxidant activity was determined by the ABTS and DPPH methods, where Taezny rubiny was most prominent. A high correlation between total phenolics and ascorbic acid content, and antioxidant activity was reported [102]. *V. opulus* (Latvia) fruits and pomace were subjected to supercritical carbon dioxide extraction (SFE-CO2) with different conditions to obtain the optimal lipophilic fraction. The antioxidant potential of *V*. *opulus* SFE-CO2 extracts was assessed by the ORAC method. The antioxidant capacity values of the washed pomace, unwashed pomace, and dried berry extracts, at the highest yield (optimal SFE-CO2 conditions), were 65.3 ± 1.8, 74.3 ± 2.2, and 142.4 ± 3.6 *μ*mol TE/g, respectively. It can be concluded that the dried berry extracts were twice as higher in the antioxidant activity expression in comparison to the pomace extracts [125].

Çanga and Dudak [126] implemented cellulose acetate/gum Arabic fibers loaded with a *V. opulus* (Turkey) fruit extract. Within the examination of the materials, they tested the antioxidant activity of the loaded fibers and observed high values of DPPH radical inhibitions ranging 56-59% and 55–58% at 4°C and 25°C, respectively.

These free radical inhibitions were more effective than the free *V. opulus* fruit extract (40% and 34%, respectively). Barak et al. [98] studied the difference in the antioxidant potential of Turkish *V*. *opulus* methanol, and water fruit extracts before and after *in vitro* gastrointestinal human digestion. Antioxidant effects of the extracts were assessed in the following phases: nondigested, postgastric, colon available, and serum available, by different methods (N,N-dimethyl-p-phenylendiamine-DMPD, cupric reducing antioxidant capacity (CUPRAC), DPPH, FRAP, and total antioxidant capacity). The methanol extract was superior in antioxidant expression than the aqueous one, and the antioxidant effect decreased during the *in vitro* digestion. An interesting investigation was conducted with *V. opulus* (Turkey) fruit pomace in wheat flower cakes at concentrations of 0, 5, 10, and 15%. Cakes' total phenolic content and antiradical activity (DPPH) increased in correlation to the level of *V. opulus* fruit pomace addition (from 10.26 ± 1.5 to 76.83 ± 4.5%) [127]. The *V. opulus* (guelder rose) fruit concentrate (65%) decreased the thiobarbituric acid reactive substance (TBARS) levels in turkey meat samples during storage at concentrations of 5% and 10%, particularly after 10, 15, and 30 days. The addition of 10% concentrate to the meat significantly reduced the TBARS in comparison to control and butylated hydroxytoluene (*p* < 0.05) both under aerobic and anaerobic conditions [128].

Erdogan-Orhan et al. [129] observed the antioxidant effects of Turkish *V. opulus* and *V. lantana* ethyl acetate, methanol, and aqueous extracts from branches, leaves, and fruits. The most powerful antioxidant agents in the ferrous ion chelating capacity test were the *V. opulus* ethyl acetate leaf extract (44.62 ± 0.02% of inhibition; 2000 *μ*g/ml) and the *V. lantana* ethyl acetate fruit extract (58.72 ± 1.00% of inhibition; 2000 *μ*g/ml). *V. opulus* aqueous extracts from branches and *V. lantana* methanol leaf extracts exhibited the highest effects (3.396 ± 0.01 and 3.401 ± 0.02; 2000 *μ*g/ml, respectively) using the FRAP method with chlorogenic acid as a reference. In the *β*-carotene bleaching assay, *V*. *opulus* ethyl acetate fruit extracts and *V*. *lantana* methanol leaf extracts were significant antioxidant agents with coefficients of 60.5 ± 1.36 and 79.50 ± 1.76, respectively, at 2000 *μ*g/ml. The authors noticed that a higher total phenolic content in the tested extracts usually indicates greater antioxidant effects [129]. Both *Viburnum* species were also studied by Altun et al. [41]. The antioxidant effects of different water extracts (branches, fruits, and leaves) were assessed using the DPPH and superoxide anion scavenging methods. Branch extracts successfully reduced the effects of the superoxide anion (IC_50_ = 3.7 and 3.1 mg/ml, respectively). On the contrary, the extracts produced a positive antiradical effect on the DPPH radical with various inhibition values, especially the *V*. *opulus* branch extract with IC_50_ values at 0.014 mg/ml [41].

Interestingly, during autumn migration birds select quality fruits rich in anthocyanins, phenolic profile, and strong antioxidant activity. Bolser et al. [130] revealed that birds preferred *V*. *recognitum* and *V*. *dentatum* fruits which have the highest total antioxidant content. Serteser et al. [131] investigated the antioxidant properties of selected wild-growing plants in Turkey. Among them, the *V. lantana* methanol fruit extract proved to be a moderate antioxidant agent in the DPPH method (EC_50_ = 1.523 mg/mg DPPH), the Fe^+2^ chelating assays (39.43 ± 2.69%), and through H_2_O_2_ inhibition (43.37 ± 2.86%). *V*. *lanata* buds, macerated in a glycerin-ethanol solution (as part of a gemmotherapy preparation for respiratory diseases), showed the weakest antiradical effect in the DPPH test (30.08 ± 2.14 *μ*g/ml) in comparison to other herbs (buds of *Betula pubescens*, *Ribes nigrum*, *Carpinus betulus*, and offshoots of *Rosa canina*). *V*. *lanata* buds were the poorest in total phenolic and flavonoid, caffeic, and chlorogenic acid contents [132]. Iranian *V. lantana* methanol leaf extract was evaluated by the DPPH method and IC_50_ value of 52 *μ*g/ml, demonstrating a great antioxidant capacity. This extract contained two chalcone glycosides (trans-3-ethoxy-4-O-(glucopyranoside)-2′, 3′, 4′, 5′, 6′-pentahydroxy chalcone and trans-3-methoxy-4-O-(glucopyranoside)-2′, 3′, 4′, 5′, 6′-pentahydroxy chalcone) isolated for the first time from *V. lantana* leaves [133].

The GC-MS data for the methanol soluble fractions of *V. sargentii* extract (originating from the Republic of Korea) highlights the presence of guanosine, levoglucosan, vitamin E, stigmast-5-en-3-ol, and stigmata-5,24(28)-dien-3-ol. Patil et al. [134] showed a significant antioxidant potential of the extract in a concentration-dependent manner for all three methods. The extract produced a strong antiradical effect in the DPPH assay with an IC_50_ value of 15.33 ± 0.58 *μ*g/ml. For both remaining methods, the extract had an electron-donating capacity which reflected its reducing power to change the ferric (Fe^3+)^ to the ferrous (Fe^2+^) form and Mo (VI) to Mo (V), respectively [134]. *V*. *nervosum* roots, essential oil, and extracts showed strong antiradical effects in DPPH system. Methanol and ethanol extracts (100% and 80%) were more potent with IC_50_ = 22.97 ± 0.38-25.65 ± 1.02 *μ*g/ml than essential oil (IC_50_ = 33.32 ± 0.67 *μ*g/ml). Additionally, the inhibition capacity of the extracts was greater in a linoleic acid system, where lipoperoxidation inhibition ranged from 47.67 ± 0.87 to 69.25 ± 1.67% in comparison to oil 44.03 ± 0.96% where the dominant compounds were *α*-eudesmol, caryophyllene oxide, linalool, spathulenol, and ledene Awan et al. [135]. Fu et al. [136] conducted an antioxidant potential screening of edible fruits from South China. They reported that *V*. *sempervirens* (in particular *V. fordiae*) fruits produced great total antioxidant effects estimated by FRAP and TEAC. However, it is important to note that the *V*. *sempervirens* nonpolar fruit fraction showed greater effects as compared to *V. fordiae*, which polar fruit fraction was more active. The authors also noticed strong correlations of antioxidant expression with total phenols [136]. Leiner et al. [137] evaluated the antioxidant capacity of Alaskan berries by the ORAC method and revealed an excellent effect of *V. edule* fruits (117 mmol of TE/g), after *Vaccinium vitis-idaea*. Antioxidant potential was also confirmed for *V. dilatatum* leaves and fruits [138, 139]. Iwai et al. [138] from Japan examined *V. dilatatum* juice (fruit squeezing solution) claiming its strong antioxidant activity. The *in vitro* antioxidant potential of the *V. dilatatum* sample was measured by the XYZ-dish and electron spin resonance (ESR) method. In the XYZ-dish technique, the tested sample expressed effective activity against OH· (10.163 ± 2.376 units/ml), as opposed to its antiperoxide effect (H_2_O_2_) (0.529 ± 0.127 units/ml). The activity of the *V. dilatatum* juice to scavenge OH· radicals, measured by the ESR method and represented as DMSO equivalent concentration, was also strong (0.937 ± 0.176 mM DMSO eq/ml) [138]. Three Indian *Viburnum* species and their methanol leaf extracts were investigated in a DPPH test, a nitric oxide (NO) scavenging test, and an assay of reduced glutathione and ferrous sulphate-induced lipid peroxidation. Ponnudurai et al. [140] concluded that these extracts could be effective antioxidants in the following order: *V. erubescens*>*V. coriaceum*>*V. punctatum*. *V. awabuki* (originating from China) and its extracts exhibited exceptional activities. The ethyl acetate-soluble fraction of the crude methanol-chloroform extract was dominant in antioxidant effects in DPPH radical inhibition (1000 *μ*g/ml = 61.88 ± 0.23%) and reducing power activity (1000 *μ*g/ml = absorbance of 0.287 ± 0.006), while the basic crude extract was prominent in the hydroxyl radical-scavenging activity test (100 *μ*g/ml = 71.26 ± 0.38%). On the contrary, other *V. awabuki* extracts: petroleum ether-soluble fraction, n-butanol-soluble fraction, and aqueous residue, were significantly weaker [141]. Abbasi [142] revealed that *V. foetens* fruit from the Himalayan region-Pakistan represented a rich source of natural antioxidants possessing significant antioxidant effects. Compared with the water extract, the acetone extract showed higher levels of the total antioxidant capacity (84.67 ± 0.48 *μ*M AAE/100 g), DPPH antiradical capacity (84.62 ± 0.63%), and hydroxyl radical scavenging capacity (75.53 ± 0.95%). They also found significant correlations between the ascorbic acid, phenols, and metal contents with free radical scavenging activity [142]. Nine *V. tinus* extracts from Turkey were screened in detail for their antioxidant potential. Antioxidant capacities of the ethyl acetate, methanol, and water extracts of leaves, branches, and fruits were tested against DPPH, DMPD, superoxide, and NO radicals. The methanol extracts of leaves, branches, and fruits and leaves' aqueous extract exhibited a remarkable DPPH antiradical activity, over 89%. The fruits' methanol extract was prominent against the DMPD radicals (67.1 ± 0.33%), the branches' aqueous extract in NO radical quenching (near 80%), and the fruits' ethyl acetate extract was the only active in the superoxide radical scavenging test (38.4 ± 1.01%). The fruits' methanol extract displayed the strongest activity in the FRAP and phosphomolybdenum-reducing antioxidant power (PRAP) tests. In the metal-chelation capacity test, the aqueous extracts were predominant with over 60% of the activity [143]. *V. tinus* from Tunisia and its leaves' acetone extract also produced strong antiradical effects in a DPPH test with high total phenolic, flavonoid, and tannin content [144]. The Indian *V. punctatum* leaf ethanol extract was tested for its scavenging effects in a DPPH and ABTS system and total antioxidant capacity (reducing power activity) in a phosphomolybdenum assay. The extract proved to be an efficient scavenging agent with IC_50_ values of 83.29 *μ*g/ml and 92.04 *μ*g/ml, respectively, with excellent reduction effects of 65.67 ± 0.15% with the maximal concentration of 100 *μ*g/ml [145]. *V. grandiflorum*, among other wild berries of the Northwestern Himalayan region, demonstrated promising antioxidant properties. Namely, the methanol fruit and leaf extract produced an antiradical effect in a DPPH system with IC_50_ values of 294.5 and 125.82 *μ*g/ml, respectively, as well as effective chelating and reducing power [146]. Fruit, leaf, and branch extracts from *V. orientale* (traditionally used in Turkey -Anatolia) were studied for their antioxidant and neurobiological effects. The fruit and branch methanol extracts (characterized by the highest total phenolic contents) showed the highest inhibition capacity against DPPH radicals and in FRAP and phosphomolibdenum reducing antioxidant power assays. The leaf aqueous extract showed the greatest NO scavenging (75.00 ± 1.22%), DMPD scavenging (33.70 ± 1.13%), and metal-chelating (54.66 ± 3.56%) at the concentration of 2500 *μ*g/ml [106]. The aqueous extract of the aerial parts of *V. punctatum* from India showed a significant antiradical effect in a DPPH test system. The extract inhibited from 44.33 ± 0.21% to 93.65 ± 0.56% of free radicals at the concentrations from 20 to 100 *μ*g/ml Susmitha et al. [147]. The antioxidant effects of *V. odoratissimum* seed extract, from China, were analysed in superoxide radical scavenging, reducing power, and lipid peroxidation inhibition assays. The butanol-soluble fraction of the methanol-chloroform crude extract was dominant in the first two tests, which was in accordance with the total phenol and flavonoid contents. The authors observed that the petrol ether-soluble fraction had the highest antilipoperoxidant activity and that the high temperature was not effective for the lipid peroxidation inhibition [148]. *V. mullaha* (India) acetone fruit extracts showed prominent antioxidant activities *in vitro* assays: ABTS, DPPH, superoxide anion, and linoleate peroxyl radicals scavenging and ferric reducing except ferrous metal chelating activity. The HR-LC-MS analysis detected 15 phenolic compounds: chlorogenic acid, acetyl salicylic acid, dihydroquercetin, dihydrorobinetin, dihydromyricetin, 2-isoprenylemodin, rutin, cosmosiin hexaacetate, pectolinarin, eriodictyol, iriginol hexaacetate, theaflavin, epicatechin pentaacetate, lomatin, and peucenin [149]. Methanol, ethyl acetate, and water extracts made of *V. grandiflorum* (Pakistan) stem exhibited antiradical activity in a DPPH assay. The water extract had the highest potential (IC_50_ = 255 *μ*g/ml) followed by ethyl acetate (IC_50_ = 322 *μ*g/ml) and methanol (IC_50_ = 742 *μ*g/ml) extracts [39].

#### 4.1.2. *In Vivo* Studies

*In vivo* studies of the antioxidant effects of *Viburnum* species are not as extensive as *in vitro* studies. The literature refers to several experimental studies which mainly included *V. opulus* and *V. dilatatum*.

The *V. opulus* (Turkey) fruit methanol extract showed protective effects against ischemia/reperfusion- (I/R-) induced oxidative stress during lung transplantation in rats, presumably due to its antioxidant effects and ability to neutralize free radicals. Namely, the treatment with the extract (200 mg/kg, intraperitoneally) significantly increased the levels of the antioxidant system (superoxide dismutase (SOD), glutathione peroxidase (GPx), catalase (CAT), and total glutathione) and repaired the total antioxidant plasma status of rats (69.59 ± 8.9 *μ*mol Trolox eq/mg protein) compared to the untreated group (43.02 ± 4.75 *μ*mol Trolox eq/mg protein). In addition, the extract reduced the malonyldialdehyde (MDA) and protein carbonyl levels. It is considered that malic, caffeic, quinic, coumaroyl-quinic, and chlorogenic acid, as well as particular caffeic acid, derivatives are responsible for the antioxidant effects previously described [150]. The impact of *V. opulus* water fruit extract (also originating from Turkey) on testicular and epididymal rats tissue treated by i.p. injection of taxane-based chemotherapeutics was investigated by measuring the lipid peroxidation level and antioxidant activities. Docetaxel and paclitaxel imbalanced an oxidant/antioxidant system, which was repaired with an oral dose of 100 mg/kg of the extract. MDA levels were significantly lower in rats' testis and epididymis while the levels of superoxide dismutase, glutathione peroxidase, and catalase were increased. The authors identified several compounds in the extract by GC-MS (*α*- and *β*-pinene, butanoic acid, DL-limonene, *α*-terpineol, and germacrene D) [151]. Furthermore, the lyophilized *V. opulus* juice and the commercial lyophilized *V. opulus* juice showed a significant antiurolithiatic activity in rats (100 mg/kg) compared to the Cystone standard. Ilhan et al. [152] attributed this effect to their antioxidant and diuretic activity and the inhibitory effects on the oxalate levels. The antioxidant action was estimated by measuring TBARS, total thiols, and glutathione in kidney tissues. TBARS levels were significantly reduced, after administration of *V. opulus* juices, with increased levels of total thiols and glutathione [152]. *V. opulus* proanthocyanidins produced gastroduodenoprotective effects against water immersion and restraint stress in rats, improving the levels of antioxidant enzymes' superoxide dismutase, catalase, and gluthatione peroxidase and decreasing the MDA content. Proanthocyanidins, as *V. opulus* extract, were intragastrically administered in three doses at 25, 50, and 75 mg/kg body weight [40].

*V. dilatatum* juice showed an inhibitory effect on gastric ulcer formation and oxidative damage caused by water immersion restraint stress in rats. The concentrations of lipid peroxides, assessed by TBARS, in the plasma, liver, and stomach were significantly lower compared with the group treated with water [138]. The same Japanese researchers tested the *V. dilatatum* crude fruit extract, proving ineffective for preventing oxidative injury induced by water immersion restraint stress. The extract improved the lipid peroxidation in the plasma, liver, and stomach but had weak effects on the enzymatic activities (superoxide dismutase, catalase, and gluthatione peroxidase). However, ferrous ascorbate-induced oxidation in hepatic homogenate of rats was inhibited. According to the results, *V. dilatatum* fruits cannot induce antioxidant enzymes and its absorbed antioxidant components have a direct effect on the oxidative injury in the body [153]. In addition, the *V. dilatatum* crude fruit extract was subjected to the experiment with streptozotocin-induced diabetic rats. The animals received *V. dilatatum* extract solution (16.8 mg/ml) for 10 weeks. TBARS levels in the plasma, erythrocytes, liver, kidney, and pancreas were significantly reduced together with plasma glucose levels. The extract contained two cyanidin glycosides, two chlorogenic acids, and quercetin. It is suggested that cyanidin 3-sambubioside is crucial for the physiological effects of *V. dilatatum* fruit, given the strong antioxidant nature of the compound [70, 154].

The leaf methanol extract of *V. tinus* from Egypt produced a significant effect on serum lipid peroxides (measuring of thiobarbituric acid-reactive substance) and nitric oxide levels (Griess reaction) with the dose of 50 mg/kg i.p. on CCl4-induced hepatotoxicity in rats, although not at lower doses of 25 mg/kg [155].

### 4.2. Antimicrobial Activities of Viburnum Plants (*In Vitro* Studies)

During the last two decades, antimicrobial activity of plant species of the genus *Viburnum* L. has been extensively studied. Antimicrobial activity of the essential oils from the air-dried whole plants of *V. opulus*, *V. lantana*, and *V. orientala* were tested against the bacteria *Escherichia coli*, *Klebsiella pneumoniae*, *Pseudomonas aeruginosa*, *Enterococcus faecalis*, *Staphylococcus aureus*, *Bacillus cereus*, and the fungus *Candida tropicalis*. The oils were at a maximum concentration of 250, 500, and 1000 *μ*g/ml in hexane, respectively. The activity was tested using the agar dilution MIC assay. The oils of *V. lantana* and *V. opulus* showed no activity against the microorganisms tested. The essential oil of *V. orientale* showed a weak antibacterial activity against Gram-positive bacteria such as *E. faecalis*, *S*. *aureus*, and *B. cereus* [83]. In another study, the essential oil of *V. betulifolium* was analysed and tested for antimicrobial activity using microdilution assay of human pathogenic bacteria and yeast. *V. betulifolium* is an evergreen shrub widely distributed throughout the Yunnan Province and southwestern parts of China. The main essential oil constituents of the species are phytol (9.8%), trans-b-damascenone (5.9%), *α*-cadinol (5.7%), *γ*-cadinene (5.6%), *Δ*-cadinene (5.3%), methyl pentanoate (4.6%), and tetradecanal (3.8%). The oil showed strong antimicrobial activity against both Gram-positive and Gram-negative bacteria and the yeast, the effect being more significant against Gram-positive than Gram-negative bacteria. Positive inhibitory activity was shown by *Pseudomonas aeruginosa* (MIC 125 *μ*g ml^−1^) and *Candida albicans* (MIC 62.5 *μ*g ml^−1^) [156]. Awan et al. [135] investigated antimicrobial activities of *Viburnum nervosum* root essential oil and several methanolic and ethanolic root extracts. *V. nervosum* is a large deciduous precocious shrub, 2-3 m tall with stiff stout branches. In Kashmir's traditional medicine, it is used as an astringent and emmenagogue, as well as for the treatment of acute furunculosis. Bergenin, a trihydroxybenzoic acid glycoside, was isolated from its roots. This glycoside is also useful in the treatment of hypercholestraemia, kidney stones, fever, diarrhea, and pulmonary infection. The essential oil of the root possesses carminative, insecticidal, antiseptic, and disinfectant properties. The main compounds of the root oil are eudesmol (30.3%), caryophyllene oxide (17.0%), spathulenol (10.7%), and linalool (12.65%). The essential oil and root extracts of *V. nervosum* were tested against Gram-positive bacteria (*Staphylococcus aureus* and *Bacillus subtilis*), Gram-negative bacteria (*Escherichia coli* and *Pasteurella multocida*), and pathogenic fungi (*Aspergillus niger*, *Aspergillus flavus*, *Fusarium solani*, and *Rhizopus solani*) with the disc diffusion method. The oil showed maximum activity against *B. subtils*, *A. niger*, and *R. solani*. However, no activity was observed by the extracts Awan et al. [135]. Nonetheless, the chemical composition and antimicrobial activity of *Viburnum* species essential oils were examined, neither were the antimicrobial activities of the dominant compounds investigated.

Bibi et al. [157] tested the antibacterial activity of the methanolic dry extract of *Viburnum foetens* (20 mg/ml), with an agar well diffusion method, against *Bacillus subtilis*, *Micrococcus leuteus*, *Salmonella setubal*, *Salmonella aureus*, and *Pseudomonas picketii*. All bacteria tested were sensitive to the extract. *S. setubal* was the most sensitive bacteria. The extracts of *V. foetens* were subject of another study. Awan et al. [158] examined four different extracts of *V. nervosum* and *V. foetens* leaves for antibacterial activities against eight different bacteria (*Staphylococcus aureus*, *Bacillus subtilis*, *Salmonella typhi*, *Pseudomonas aeruginosa*, *Klebsiela pneumoniae*, *Proteus vulgaris*, *Citrobacter freundii*, and *Streptococcus pneumoniae*) using the disc diffusion method. The study concluded that the ethanolic extract was the most effective, contrary to that of the petroleum ether extract. The ethanolic and methanolic extracts showed prominent activities against all tested bacteria, in comparison to the chloroform extract which had moderate activity. Turker et al. [159] used the same antibacterial activity evaluation method by analysing the antimicrobial activity of the *V. lantana* extracts obtained from dry and fresh fruits (water and ethanol) against Gram-positive bacteria (*Streptococcus pyogenes*, *Staphylococcus aureus*, and *Staphylococcus epidermidis*) and Gram-negative bacteria (*Escherichia coli*, *Pseudomonas aeruginosa*, *Salmonella typhimurium*, *Serratia marcescens*, *Proteus vulgaris*, *Enterobacter cloacae*, and *Klebsiella pneumoniae*). Gram-positive bacteria were more susceptible to the inhibitory effects of the plant extracts than the Gram-negative bacteria. The extracts of *V. lantana* fresh fruits exhibited antibacterial activities. The inhibition capacity of the hot ethanolic extract was greater than the cold ethanolic extract against *S. aureus*, *S. epidermidis*, and *S. pyogenes.* Both *S. marcescens* and *P. aeruginosa* were resistant to all examined fruit extracts.

Eryilmaz et al. [160] also studied the antimicrobial activity of *V. lantana*, together with *Viburnum opulus* L., *V. orientale* Pallas, and *V. tinus* L. against *Staphylococcus aureus*, *Bacillus subtilis*, *Escherichia coli*, *Pseudomonas aeruginosa*, *Klebsiella pneumoniae*, and *Candida albicans*. The disc diffusion and tube dilution techniques were used to determine the activities of the extracts. Ethanolic and water plant extracts of leaf, stem, and fruit were used in the experiment. Ethanolic extracts from all analysed species showed antimicrobial activity against all the tested microbes. Water extracts were either weak or not effective against tested microorganisms. The antimicrobial activity of the dried fruit methanolic extract of *V. opulus* was also analysed by another study with an agar diffusion method [119]. The study analysed ten microorganisms (*Aeromonas hydrophila*, *Bacillus cereus*, *Enterobacter aerogenes*, *Escherichia coli*, *Klebsiella pneumoniae*, *Proteus vulgaris*, *Pseudomonas aeruginosa*, *Salmonella typhimurium*, *Staphylococcus aureus*, and *Yersinia enterocolitica*). A 15% concentration extract completely inhibited the growth of all analysed bacteria. The same plant species have shown the capacity to reduce the potential of *Staphylococcus aureus* and *S. epidermidis* to colonize inert substratum and form biofilms [121]. Similarly, fruit juices and ethanolic extracts of *V*. *opulus* genotypes were tested against ten Gram-positive and Gram-negative bacterial cultures and nine yeast strains. The fruit juices showed greater antibacterial activity compared to the ethanol extracts. The most effective antibacterial activity was exhibited by the juices against *Salmonella typhimurium*, *Salmonella agona*, and *Listeria monocytogenes*. The fruit juices and ethanol extracts showed weak or no activity on the yeast strains [161]. Antimicrobial activities of fruit juices of six *V. opulus* genotypes were evaluated by Česoniene et al., [42], using the agar well diffusion method against ten Gram-positive and Gram-negative bacteria and seven strains of yeast. The juices strongly inhibited the growth of Gram-negative (*S. typhimurium* and *S. agona)* and *Gram-positive (S. aureus*, *L. monocytogenes*, and *Enterococcus faecalis*) bacteria. As previously reported, the effect of the juices on the yeast was low or lacking [42]. Up to Česonienė's studies, most *Viburnum* plant extracts had presented the highest effect on Gram-positive bacteria strains and some yeasts. However, antimicrobial analysis of the juices has shown that they can be used to fight Gram-negative microorganisms. Differences in antimicrobial activity are most likely due to the chemical composition of the extracts and juices. Therefore, further research of *Viburnum* species should connect the chemical composition with the antimicrobial activity.

Paulauskas et al. [162] went one step further. They analysed the antimicrobial activity of unripe mashed berries and ripe berry juice of *V. trilobum* Marshall, *V. sargentii* Koehne, and *V. opulus* cultivar “Leningradskaya Otbornaja.” The unripe berry mass and ripe berry juices both significantly influenced the bacteria. The unripe berry mass manifested greater antibacterial activity, similarly, on both the Gram-positive and Gram-negative bacteria. *Micrococcus* sp. and *S. aureus* were the most sensitive bacteria to the mashed berries and all analysed juices. *Viburnum* juice impacted the microscopic fungi the least.

Methanolic extract of *V. cotinifolium* leaves from Pakistan was tested against four Gram-positive bacteria, five Gram-negative bacteria, and ten fungal strains with the agar diffusion method. The extract demonstrated maximum activity against *Aspergilus flavus* and *A. fumigates*. The extract also showed positive antimicrobial activity against *A. niger*. The *V. cotinifolium* extract showed the most effective activity against *Enterococcus faecalis* and *Enterobacter coccus*. As can be observed, the extract of *Viburnum* species was also effective against Gram-negative bacteria [163]. This indicates that *V. cotinifolium* leaf methanolic extract has great potential as a natural antimicrobial agent. Hence, chemical analyses of the extract should be carried out and associated with antimicrobial activity. This connection is supported by the research carried out by Roy [164], which proves that the methanolic extract (and fraction) from the whole plant of *V. foetidum* exhibit a significant antimicrobial activity against Gram-positive and Gram-negative bacteria strains, as well as significant antifungal activity. The ethyl acetate (EA) fraction from the methanolic extract exhibited the highest antimicrobial potential. Agrobacterium species were most susceptible to the EA fraction of the extract, and clearly, the EA fraction differs in its composition from its counterparts. Unfortunately, detailed chemical analyses of the extracts and/or fractions have currently not been carried out.

### 4.3. Anti-Inflammatory Activities of Plant Species of the Genus *Viburnum* L. (*In Vitro* Studies/*In Vivo* Studies)

Inflammatory diseases are usually treated by steroid drugs, nonsteroidal anti-inflammatory drugs, and immunosuppressant. Although the effects of these drugs have been proven, their side effects are not negligible. The usage of these drugs is often associated with bleeding gastrointestinal and peptic ulcers [165]. In search for new harmless drugs, scientists are once again turning to medicinal plants. Among these plants, the species of the genus *Viburnum* are of interest. For that purpose, anti-inflammatory activities of *V. lantana*, *V. trilobum*, *V. pichinchense*, *V. sargentii*, *V*. *fordiae*, and *V. opulus* were investigated [78, 166–170].

The bark of this species has been used in Turkish traditional medicine as a rubefacient and analgesic [41]. *Viburnum lantana* L. leaf water extract was investigated for anti-inflammatory activity, in rats with a carrageenan-induced rat paw edema test. The anti-inflammatory activity of the extract at doses of 100 and 200 mg/kg has been low as compared to indomethacin [169].

*Viburnum trilobum* Marshall (American highbush cranberry) is widely used in traditional medicine as it displays an anti-inflammatory and antidiabetic effect, sometimes used to improve lipid metabolism. The bark can act as a sedative and pain reliever. Due to the high content of ursolic acid, which expresses anti-inflammatory properties, the bark acts as an anti-inflammatory agent. This effect is proven in a RAW 264.7 macrophage cell system. All fractions of the *V. trilobum* ethanolic extract significantly inhibited the levels of IL-1*β*, IL-6, and TNF*α* [168].

*Viburnum pichinchense* Benth also displays anti-inflammatory properties. The anti-inflammatory effects of the methanol extract were demonstrated using LPS-stimulated macrophages and HCl/EtOH-induced gastritis model mice. The extract expresses anti-inflammatory activity by targeting NF-*κ*B and caspase-11 noncanonical inflammasome pathways in macrophage-mediated inflammatory responses [167].

Leaves, stems, and fruits of the plants have been used in traditional folk medicines as therapeutic agents, as styptics and analgesics, to treat boils, rheumatoid arthritis, traumatic injuries, ringworm, skin itching, and coughs [170]. In the last two decades analgesic, anti-inflammatory, and hepatoprotective activities of its methanol extract were confirmed. The butanol fraction of the methanolic extract showed the highest activity on inflammatory reactions [170].

*Viburnum fordiae* Hance, a small tree widely distributed in the south of China, has been used in traditional Chinese medicine for centuries to treat rheumatic arthralgia and allergic dermatitis. Recent studies have reported a new, unusual ɣ-lactone, obtained from the aerial parts of these plants, capable of expressing an *in vitro* anti-inflammatory effect *vitro* [78].

*V. opulus* is well known as a medicinal and horticultural plant with a dietary value. Its fruits have been used in traditional medicine to cure pulmonary, stomach, cardiovascular, and kidney diseases, as well as for the treatment of cramps, diabetes, bleeding, coughs, and colds. Arginase activity and arterial vasodilation of the plant extract have also been proven [100, 101, 104, 171–173]. The anti-inflammatory activity of *V. opulus* water leaf extract was conducted in rats by a carrageenan-induced rat paw edema, test at doses of 50, 100, and 200 mg/kg, i.p., proving the extract had no anti-inflammatory effect at these doses [166].

### 4.4. Cytotoxic Activities of *Viburnum* Plants (*In Vitro* Studies)

For centuries, herbs and plants have had a role in the treatment of various forms of tumors as have also shown to reduce the risk of cancer development or serve as a treatment for different types of cancer [174]. *Viburnum* species and their products have been extensively studied for their cytotoxic properties, being promising anticancer agents. To date, the most studied species in this regard is *V. opulus*.

Sauter and Wolfensberger [175] were the first to report the cytotoxic activity of *Viburnum* extracts. Aqueous fruit extracts of *V. opulus* and *V. lantana*, from Switzerland, showed no cytotoxic activity on BT 20 breast cancer cells within 72 h of incubation. Similarly, further research revealed no cytotoxicity of *V. opulus* extracts. An Indian aqueous bark extract was tested for its cytotoxic effect using a simple bioassay, brine shrimp lethality test, but no remarkable effect was observed [3]. The same findings were reported for *V. opulus* seed extracts by Cantrell et al. [176]. Russian *V. opulus* fruits and its ethanol extract exhibited low cytotoxicity, suppressing cell growth at concentrations above 200 *μ*g/ml [3].

On the contrary, some authors consider *V. opulus* as an effective cytotoxic agent. Laux et al. [177] investigated the aldehyde fraction of the *V. opulus* chloroform-methanol fruit extract (Canada) for the cytotoxic effect on human gastric carcinoma cells. The fraction containing (E) 2-hexenal, (Z) 2-decenal, 2,4–decenal, (E) 2-octenal, and 2-undecenal produced a direct antiproliferative effect on the growth of the carcinoma cells with death at a concentration of 27 *μ*M [177]. The methanol and acetone extract, juice, and juice after extraction to the solid phase of *V. opulus* from Poland showed cytotoxic activity against human breast (MCF-7) and cervical (HeLa) cancer cell lines. The strongest toxic agent towards both cell lines was observed for the juice obtained after purification with IC_50_ values of 63.541 and 19.380 *μ*g/ml for HeLa and MCF cell lines, respectively [178]. According to IC_50_ values (250-450 *μ*g/ml), the cytotoxicity of the same origin *V. opulus* extracts against Caco-2 cells, measured with the PrestoBlue assay, were in the following order: phenoli- rich fraction from fresh juice>methanol-acetone extract from pomace>acetone extract from pomace>fresh juice. This effect could be attributed to the highest content of phenolic compounds (flavanols, falvonols, hydroxycinnamic acids, and anthocyanins) [99]. The commercial *V. opulus* juice from Turkey produced a cytotoxic effect against Caco-2 (human colon adenocarcinoma) and HeLa cells but was not active in a test using A549 (human type II lung epithelium) cells over a 72-hour period with the concentrations of 10-80 *μ*g/ml. Futhermore, *V. opulus* juice caused no significant decrease in the viability of MDCK (Madin Darby Canine Kidney) and HUVEC (Human umbilical vein endothelial cells) normal cell lines [179].

Antitumor activities of water and ethanol extracts prepared using the hot and cold procedures of fresh and dried *V. opulus* and *V. lantana* fruits (Turkey) were tested with the potato disc tumor induction method. *V. opulus* was more active with the inhibition of 61.9-100%. Both water extracts from dried fruits were the most effective in the assay. Among V. lantana extracts, hot water and ethanol extracts were the most effective, 90.5% and 95.2%, respectively [159]. The same technique was used to assess the antitumor activity of aqueous, ethanol, and methanol *V. lantana* (Turkey) leaf and fruit extracts. The methanol extract was the most effective with 100% of tumor inhibition, followed by the ethanol (90.9%) and the aqueous one (86.4%) [180].

The researchers from Pakistan, Shah et al. [37], conducted an identical assay for cytotoxicity with *V. grandiflorum* methanol extract and its n-hexane, chloroform, ethyl acetate, and n-butanol fractions. The chloroform extract was more active, expressing a strong ability to kill brine shrimp (EC_50_ = 107.45 *μ*g/ml). The methanol extract of *V. grandiflorum* from China exerted a strong impact on lung cancer cells H1650, HCC827, and H1299 by decreasing their viability in a concentration- and time-dependent manner. The viability of the H1650, HCC827, and H1299 cells decreased to 34%, 31%, and 29%, respectively, after three days of treatment with the extract. A detailed analysis found that the viability of cells was inhibited by the apoptosis activation through a caspase-dependent pathway [181].

*V. punctatum* from India was tested in several studies. A *V. punctatum* methanol extract made from aerial parts displayed cytotoxic activity against human liver cancer cells (HepG2) with CTC50 (cytotoxicity 50%) values of 205.8 ± 1.92 *μ*g/ml by the 3-(4,5-dimethylthiazol-2-yl)-2,5-diphenyltetrazolium bromide (MTT) test and against human laryngeal epithelial carcinoma (Hep2) with CTC50 value 197.3 ± 2.89 *μ*g/ml [182]. *In vitro* anticancer activity of aerial parts of *V. punctatum* was tested using chloroform and methanol extracts and the HCT 15 cell line (human colon carcinoma). Maximal concentrations of the extracts (400 *μ*g/ml) inhibited 63.93 ± 2.76% and 80.16 ± 2.13% of cell viability, respectively [183]. *V. punctatum* chloroform and methanol extracts expressed a hepatoprotective activity *in vitro* protecting the Chang liver cells against CCl4-induced toxicity in the MTT test. The cell viability ranged from 62% to 84% at concentrations of 200-400 *μ*g/ml, with the methanol extract being more effective [184]. The ethanol extract of *V. punctatum* leaves showed anticancer activity against MCF-7 in MTT test with IC_50_ of 56.73 *μ*g/ml [185].

The *V. foetens* (Pakistan) methanol crude extract and fractions showed a significant anticancer effect against the breast cancer cell line MCF-7. The crude extract was active 90.5% at 200 *μ*g/ml, while the fractions were less effective at the same concentrations. The highest MCF-7 cell line inhibition percentage was reported for the methanol (83%), followed by the chloroform 55.5%, the hexane 25.11%, and water (2%) fractions [157]. The crude methanol extract and fractions of *V. foetens* (Pakistan) were evaluated against MCF-7, MDA-MB-468, and Caco-2 cancer cell lines by the MTT test and NRU (neutral red uptake) assay. The crude extract inhibited the cancerous cell growth in a dose-dependent manner. The ethyl acetate fraction significantly reduced Caco-2 cells (93.44%) growth in the MTT test. The methanol and ethyl acetate fractions decreased 99% and 96% cell growth of MCF-7 and Caco-2 cell lines, respectively, in the NRU assay. Also, ethyl acetate fraction of the *V. foetens* extract exhibited a considerable inhibition of MDA-MB-468 cells in both used assays. Other fractions (chloroform, hexane, and aqueous) produced a weaker effect on cancer cell proliferation [186]. Methanol leaf extract of *V. dilatatum* (Korea) produced a cytotoxic effect on MCF-7 human breast cancer cells with IC_50_ of 139 ± 16 *μ*g/ml. The authors reported that this effect could not be considered strong, according to the screening program of the National Cancer Institute, USA, which recommends IC_50_ under 20 *μ*g/ml to be the effective cytotoxic agent [139]. Roy [164] reported significant lethality in a brine shrimp cytotoxicity assay for the *V. foetidum* crude methanol extract and its petroleum ether and n-hexan fractions with LC50 of 39.81, 25, and 25 *μ*g/ml, respectively, while LC50 for standard vincrinstine sulphate was 10.44 *μ*g/ml. The Colombian *V. cornifolium* leaf dichlormetan extract showed high cytoxicity tested on the V79 cell line (Chinese hamster lung fibroblasts) with IC_50_ of 25 *μ*g/ml [187]. Ponnudurai et al. [188] (India) tested methanol leaf and chloroform root extracts of *V. coriaceum* and *V. erubescens* for their bacterial strain-based cytotoxicity (*E. coli* AB 1157 strain), using the MTT method (MCF-7 breast cancer cell lines and HeLa cervical cell lines). All extracts, except the chloroform root extract of *V. erubescens*, showed an effect on the bacterial strain-based carcinogenicity. IC_50_ values in the MTT test, only determined for the *V. coriaceum* extracts, indicated a moderate anticancer activity (over 500 *μ*g/ml for MCF-7 cells and 300 *μ*g/ml HeLa cells) [188]. Calderón-Montaño et al. [189] reported cytotoxic effects for the Spanish V. tinus water fruit and leaf extracts in the MTT test. The fruit extract was more potent in the inhibition of proliferation of A549-human lung adenocarcinoma cells and MRC-5-human lung fibroblastic cells with IC_50_ of 26.6 ± 6.5 and 65.4 ± 8.6 *μ*g/ml, respectively. Methanol extracts of Chinese *V. odoratissimum* wood and bark successfully inhibited melanin biosynthesis and cell proliferation of B16 melanoma cells at 100 and 50 *μ*g/ml, respectively [190].

### 4.5. Anticancer Effects of Viburnum Plants *In Vivo*

The anticancer effect of *V. opulus* juice was previously reported on Ehrlich ascites carcinoma cells [26]. However, Ceylan et al. [191] from Turkey investigated *V. opulus* juice for its antitumor potential in an in vivo experiment with experimental Balb/c mice. To implement tumors to mice, they applied Ehrlich ascite carcinoma (EAC) 1 × 10^6^ cells i.p. and lyophilized *V. opulus* juice at a dose of 1000, 2000, and 4000 mg/kg. The tumor weight significantly decreased in mouse groups treated with the juice compared to the control group. The survival rate of Ehrlich ascites tumor cells was reported to be 88.72%, 69.02%, and 51.87%, respectively. The results of the *in vitro* assay indicated the cytotoxicity of the juice with the IC_50_ value of 199.58 *μ*g/ml [191]. Also, the same authors reported that gilaburu fractions below and above 50 kDa can stop the cell cycle at the G0/G1 stage and slow the cell division of the Ehrlich ascites tumor [123].

Ulger et al. [192] (Turkey) experimented with Balb-c male mice to study the effect of *V. opulus* juice on colon tumorogenesis induced with 1,2-dimethylhydrazine (DMH). All groups treated with DMH developed colon tumors as observed by histogenesis. However, mice that received the juice showed a reduced number of tumor lesions, as well as the incidence of invasive carcinoma, as compared to untreated mice. The authors concluded that *V. opulus* juice could be useful at the initiation stage and prevention of colon cancer.

### 4.6. Other Health-Promoting Effects

A 1000 mg *Viburnum opulus* dose and diclofenac *on-demand* were administered, orally, to 53 patients with urethral stones < 10 mm, in comparison to 50 patients receiving only diclofenac *on-demand.* It was observed that the expulsion of the stones was greater, and the passage time was faster in the treatment with *V. opulus*. The demand for diclofenac was lower compared to patients who received only diclofenac [193]. There are no clinical trial reports for the premenstrual syndrome [111], only the folk medicine use, as antispasmodic, in menstrual cramps, dysmenorrhea, and miscarriage prevention [194]. No side effects or reports regarding safety were found [194, 195].

## 5. Conclusions

The genus *Viburnum* includes about 200 species, distributed mainly on the Asian side Fruits, flowers, and barks of certain species are used in traditional medicine for the treatment of diseases, such as rheumatoid arthritis, cough, diarrhea. They contain a plethora of biochemical compounds with health-promoting activity, including carotenoids, polyphenols, and flavonoids, which can explain the high antioxidant activity as shown by in vitro studies. Preclinical evidence supports antibacterial, anti-inflammatory, cytotoxic, and anticancer properties of certain species, such as *V. opulus*.

## Figures and Tables

**Table 1:** Ethnomedical use of genus Viburnum.

S. no.	Species name	Part used	Medicinal applications	References
1	*Viburnum grandiflorum*	Wood, leaf, flower, bark	Digestive problems, purgative, abdominal pain, diuretic, antimalarial, respiratory diseases, toothaches, yphoid, whooping cough, anesthetic	([35]; [36]; [37]; [38]; [39])
2	*Viburnum opulus*	Fruit	Gall bladder, liver disease, diuretic; bleeding, heart disease, blood pressure, coughs, cold, neurosis, diabetes	([40]; [41]; [42]; [43]; [44]; [45]; [34])
3	*Viburnum cylindricum*	Leaves	Cough, diarrhea, rheumatoid arthritis, and tumefaction Cough, diarrhea, rheumatoid arthritis, and tumefaction Cough, diarrhea, rheumatoid arthritis, tumefaction, insecticide	([46]; [30]; [47])
4	*Viburnum dilatatum*	Fruits	Spice, pickles	[48]
5	*Viburnum cotinifolium*	Bark	Hepatic and digestive problems	[47]
6	*Viburnum erubescens*	Roots, stem, leaves	Cough, insecticide	([49]; [47])
7	*Viburnum lantana*	Bark	Rubefiant, analgesic	[41]
8	*Viburnum foetens*	Whole plant, bark	Purgative, sedative, cleaning teeth “miswak”	[50]
9	*Viburnum punctatum*	—	Fever, stomach disorders	[51]
10	*Viburnum prunifolium*	Roots	Dysmenorrhea, menstrual irregularities, convulsions, hysteria, fever, palpitation, heart diseases, hysterical fits, arthritis, heart tonic, improves blood circulation	([52]; [51])
11	*Viburnum prunifolium*	—	Sedatives, muscle relaxants, cardiotonics	[53]
12	*Viburnum nervosum*	Leaf, bark, root	Purification of blood, carminative, hemorrhage, uterine disorders, asthma, furunculosis, menorrhagia	([54]; [55]; [52])
13	*Viburnum coriaceum*	Root and bark	Antispasmodic, uterine relaxant	([56]; [52])
14	*Viburnum foetidum*	Leaves, aerial parts	Menorrhagia, hypothermic, cardiovascular; uterine disorders, skin disease, emmenetic	([52]; [57])
15	*Viburnum jucundum*	—	Cancer, gastrointestinal diseases	([52]; [58])

**Table 2:** Main phytochemicals found in the species of the genus *Viburnum*.

Viburnum spp.	Phytoconstituents	Type of extract	Part of plant	Biological activities	References
*V*. *arboricolum*	Viburolide	—	Leaves and twigs	Antitumor effects	[31]
*V. ayavacense*	7,10,2′,3′-Tetraacetylsuspensolide F, 7,10,2′,3′-tetraacetylisosuspensolide F, 7,10,2′,6′-tetraacetylisosuspensolide F, 2′,3′-diacetylvalerosidate, 2′,3′-diacetylisovalerosidate, isoviburtinoside II, isoviburtinoside III, isosuspensolide E, isosuspensolide F.	—	Leaves and twigs	Antitumor effects	[31]
*V. awabuki*	6-O-methyl-6,7-dihydroxyvibsanin B, 4-hydroxyvibsanin A, 14(R^∗^),15-epoxyneovibsanin B, 14(S^∗^),15-epoxyneovibsanin B, (8Z)-neovibsanin B, 18-O-methylvibsanin C, (8Z)-vibsanin E.	—	Leaves	—	[29]
	Vibsanin G, vibsanin H, vibsanin K, vibsanin O, vibsanin P, vibsanin Q, vibsanin R, vibsanin S, vibsanin T, vibsanin U, vibsanin V, vibsanin W, furanovibsanin A, furanovibsanin B, furanovibsanin C, furanovibsanin D, furanovibsanin E, furanovibsanin F, furanovibsanin G, neovibsanin A, neovibsanin B, neovibsanin C, neovibsanin D, neovibsanin G, neovibsanin H, neovibsanin I, spirovibsanin A, 7-epineovibsanin D, 3-*O*-methylfuranovibsanin A, 7-epifuranovibsanin B, 15,18-di-*O*-methylvibsanin H, 18-*O*-methylvibsanin K, cyclovibsanin A, 15-*O*-methylcyclovibsanin A, 15-*O*-methylcyclovibsanin B, 3-hydroxy-15-*O*-methylcyclovibsanin A, 15-*O*-methylneovibsanin F, 15-*O*-methyl-14-epineovibsanin F, 15-*O*-methyl-18-oxoneovibsanin F, 2-*O*-methylneovibsanin H, 2-*O*-methylneovibsanin I, 14-epineovibsanin G, 5-epivibsanin C, 5-epivibsanin E, 5-epivibsanin H, 5-epivibsanin K, 18-*O*-methyl-5-epivibsanin K, 3-hydroxyvibsanin E, 3b,28-dihydroxyolean-12-en-1-one, 3b,28-dihydroxyolean-12-en-11-one, 13,28-epoxyolean-11-en-3-one, 6a-hydroxy-3-oxolup-20(29)-en-28-oic acid, *Ψ*-taraxasterol acetate, 6b-hydroxy-3,20-dioxo-30-norlupan-28-oic acid, 4,20-dihydroxy-3,4-secolupane 3,28-dioic acid 3-methyl ester, awabukinol, 3-hydroperoxyawabukinol, 4-hydroperoxyawabukinol, epicatechin, catechin, 7R-dihydrodehydrodiconferyl alcohol 4-*O*-*β*-D-glucopyranoside, 8R-dihydrodehydrodiconferyl alcohol 4-*O*-*β*-D-glucopyranoside, vibsanol, 9′-*O*-methylvibsanol, dihydrodehydrodiconiferyl alcohol, 3′,6'′-*O*-diacetylscopolin, 2′-*O*-acetylscopolin, 6′-*O*-acetylscopolin	—	Leaves and twigs	—	[31]
*V. betulifolium*	Viburnalloside, decapetaloside	—	Leaves and twigs	Antitumor effects	[31]
*V. chinshanence*	Lignan, chinshanol A.	—	Roots	—	[63]
*V. coriaceum*	Phytosterols, triterpenoids, phenolics, phenolic glycosides	—	Roots	—	[64]
*V. cotinifolium*	Biflavonoid	Petrol, benzene and methanol	Leaves	—	[65]
*V. cylindricum*	3-*O*-Caffeoylquinic acid methyl ester, 4-*O*-caffeoylquinic acid methyl ester, 5-*O*-caffeoylquinic acid methyl ester	—	Leaves and twigs	Antitumor effects	[31]
	2′-O-Acetylhenryoside, 2',3'-di-O-acetylhenryoside, 2′,6′-di-O-acetylhenryoside, 2′,3′,6′-tri-O-acetylhenryoside, 2′,3′,4′,6′-tetra-O-acetylhenryoside, 2-[(2,3-di-O-acetyl-beta-D-glucopyranosyl)oxy]-6-hydroxybenzoic acid, 6-hydroxy-2-[(2,3,4,6-tetra-*O*-acetyl-beta-D-glucopyranosyl)oxy]benzoic acid	Methanolic extract	Leaves and stems	—	[66]
*V. dilatatum*	Viburnol A, viburnol B, viburnol C, viburnol D, viburnol E, viburnol F, viburnol G, viburnol H, viburnol I, viburnol J, viburnol K, viburnudienone B1 methyl ester, viburnudienone B2 methyl ester, viburnenone B1 methyl ester, viburnenone B2 methyl ester, viburnudienone H1, viburnudienone H2, 2,3,4-trihydroxybutyl 6-*O*-(E)-caffeoyl- *β*-D-glucopyranoside, 2,3,4,5-tetrahydroxyhexyl 6-*O*-(E)-caffeoyl-*β*-D-glucopyranoside, arbutin, furcatin, 4-allyl-2-methoxyphenyl 6-*O*-*β*-D-apiofuranosyl(1 → 6)-*O*-*β*-D-glucopyranoside, *p*-hydroxyphenyl 6-*O*-(E)-caffeoyl- *β*-D-glucopyranoside, *p*-hydroxyphenyl 6-*O*-(E)-caffeoyl- *β*-D -allopyranoside, salidroside, 3-*O*-caffeoylquinic acid, 4-*O*-caffeoylquinic acid, dilaspirolactone, kuromanin	—	Leaves and twigs	—	[31]
	Jiamizioside E, jiamizioside A, jiamizioside B, jiamizioside C, jiamizioside D	—	Fruits	—	([67]; [68])
	Cyanidin 3-sambubioside, 5-caffeoyl quinic acid	—	Fruits	—	[69]
	Cyanidin 3-sambubioside, cyanidin 3-glucoside, quercetin, 5-O-caffeoyl-4-methoxyl quinic acid, chlorogenic acid.	—	Fruits	—	[3]
	Cyanidin 3-sambubioside, cyanidin 3-glucoside, 4-methoxy chlorogenic acid, chlorogenic acid, quercetin	—	Fruits	—	[70]
	3Z-Hexenol, l-linalool	—	Flower	—	[71]
	2-(-Glucopyranosyloxy)-benzyl 3-(-glucopyranosyloxy)-benzoate	—	Roots	—	[72]
*V. erosum*	7-*O*-Tigloylsecologanolic acid, 7-ketologanin, 7-*O*-benzoylsecologanolic acid, 7-ketologanin	Methanolic, ethyl acetate, n-butanol, water	Stems	—	[73]
	Vibruresinol, (70 R,8S,80 S)-3,50-dimethoxy-30,4,80,90 -tetrahydroxy70,9-epoxy-8,80–lignan, (+)-syringaresinol, (+)-pinoresinol, (+)-pinoresinol-4-*O*-*β*-D-glucopyranoside, herpetol, vibsanol, (-)-dehydrodiconiferyl alcohol, icariside E4, (-)-dihydrodehydrodiconiferyl alcohol	Methanolic extract	Stems	Neuroprotective activity on glutamate-induced cell death in HT22 cells	[74]
	Loganic acid, sweroside, 7-O-tigloylsecologanol, 3,7-dihydroxy-8-methyl-cyclopenta[c] pyran-4-carboxylic acid, rel-(1S,5R,9S)-9-ethenyl-1-(beta-D-glucopyrinosyloxy)-5,9-dihydro-5-{2-[(2-methylbut-2-enoyl)oxy]ethyl}-1H-pyran-4-carboxylic acid, viburnin, epi-7-O-tigloylsecologanolic acid.	Methanolic extract	Stems	—	[75]
*V. erubescens*	Phytosterols, triterpenoids, and phenolic compounds and their glycosides	—	Roots	—	[64]
	Phytosterols, triterpenoids, glycosides (saponins), phenolic compounds (flavonoids and procyanidins)	Soxhlet method	Leaves and stems	—	[76]
*V. fordiae* Hance	(7S,8R)-4-Hydroxy-3,3′,5′-trimethoxy-8′,9′-dinor-8,4′-oxyneolignan-7,7′,9-triol, (7R,8R)-4-hydroxy-3,3',5'-trimethoxy-8′,9′-dinor-8,4′-oxyneolignan-7,7′,9-triol, (7R,8R)-4-hydroxy-3,3′,5′-trimethoxy-8,4′-oxyneolignan-7,9,9′-triol-7′-one, *γ*-lactone, 3-(3,4-dihydroxyphenyl)-4-pentanolide, uvaol, 28-nor-urs-12-ene-3b,17b-diol, 2,3-O-isopropylidenyl-2a,3a,19a-trihydroxyurs-12- en-28-oic acid, erythrodiol, oleanolic acid, lupeol, megastigmadien-3,9-dione, loliolide, dehydrololiolide, 2a-hydroxycineole, (+)-isolariciresinol, umbelliferone, 3-(4- hydroxy-3-methoxyphenyl)propane-1,2-diol, 1-(4-hydroxy-3-methoxyphenyl)-1- methoxypropan-2-ol, coniferyl aldehyde, p-hydroxylcinnamaldehyde, (+)-2-hydroxy-1-(4-hydroxy-3- methoxypheny) propan-1-one, syringaldehyde, protocatechuate, 3,4-dihydroxybenzoic acid methyl ester, vanillin, p-hydroxybenzaldehyde, salicylic acid, benzyl alcohol, hydroquinone	—	Aerial parts	—	([77]; [78])
	7,8-bis-*O*-Isopropylidene-dihydroeugenol	—	Air-dried plants	—	[79]
	Fordioside, alangilignoside D, salicin, rhapontigenin	—	Leaves	—	[67]
	C-13-Norisoprenoid, alangionoside C, pisumionoside, koaburaside, 3,5-dimethoxy-benzyl alcohol 4-O-*β*-D-glucopyranoside, 3,4,5-trimethoxybenzyl- *β*-D -glucopyranoside, arbutin, salidroside, (3R,9R)-3-hydroxy-7,8-didehydro-*β*-ionyl 9-O-*α*-D-arabinopyranosyl-(1 → 6)-*β*-D-glucopyranoside, 2-(4-O-*β*-D-glucopyranosyl) syringylpropane-1,3-diol	—	Stems	—	[80]
	Norneolignan glycoside, 7-noraryl-4′,7-epoxy-8,5′-neolignan glycoside, (7R,8R)-guaiacylglycerol 4-O-*β*-D -(6-O-vanilloyl) glucopyranoside, (7S,8S)-guaiacylglycerol 4-O- *β*-D-(6-O-vanilloyl) glucopyranoside, (7S,8R)-guaiacylglycerol 4-O-*β*-D-(6-O-vanilloyl) glucopyranoside, coniferyl alcohol 4-O-[6-O- (4-O-*β*-D-glucopyranosyl)vanilloyl]-*β*-D-glucopyranoside	—	Stems	—	([77]; [78])
	Viburfordoside A, viburfordoside B, viburfordoside C, viburfordoside D, viburfordoside E, viburfordoside F, viburfordoside G, viburfordoside H, viburfordoside I. Fordiane A, fordiane B	—	Fruits	—	[81]
*V. formosanum*	Dioxatricyclodecane	Methanolic extract, ethyl acetate	Leaves	—	[82]
*V. furcatum*	Furcatoside A, furcatoside B, furcatoside C, isoquercitroside, kaempferol 3-*O*-b-d-glucopyranosyl-7-O-a-l-rhamnoside, furcatin,	—	Leaves and twigs	Antitumor effects	[31]
*V. grandifolium*	Luteolin, 3′-*O*-b-d-xylopyranosyl (1 → 2)-*O*-b-d-glucopyranoside	—	Leaves and twigs	Antitumor effects	[31]
*V. japonicum*	2′,3′-*O*-Diacetylfurcatoside C, chavicol	—	Leaves and twigs	Antitumor effects	[31]
*V. lantana*	2′-Acetyldihydropenstemide, 2′-acetylpatrinoside, 3′-acetylpatrinoside, lantanoside, dihydropenstemide, betulalbuside A.	—	Leaves and twigs	Antitumor effects	[31]
	2-Heptanone, n-heptanal, benzaldehyde, 1-octen-3-ol, 6-methyl-5-hepten-2-one, 2-pentylfuran, 2, 4 heptadienal, n-octanal, limonene, n-octanol, cis-linalool oxide, L-linalool, n-nonanal, *α*-terpineol, methyl salicylate, n-decanal, 2E, 4E-nonadienal, 2E-decanal, cinnamaldehyde, 2E, 4Z-decadienal, 2E, 4E-decadienal, *α*-cubebene, *α*-copaene, *β*-bourbonene, E-caryophyllene, *β*-copaene, geranyl acetone, *γ*-murolene, *α*-amorphene, germacrene D, *β*-ionone, *α*-muurolene, *γ*-cadinene, *Δ*-cadinene, trans-cadina-1, 4-diene, *α*-calacorene, occidentalol, E-nerolidol, spathulenol, caryophyllene oxide, salvial-4(14)-en-1-one, *γ*-eudesmol, *α*-muurolol, *β*-eudesmol, *α*-cadinol, occidenol, eudesma-4(15,7-dien-1-*β*-ol, pentadecanal, 6, 10, 14-trimethyl-2-pentadecanone, nonadecane, n-heneicosane, n-docosane, n-tricosane	Hydrodistillation	Air-dried whole plants	Antimicrobial activity	[83]
*V. luzonicum*	Luzonial A, luzonial B, luzonoside A, luzonoside B, luzonidial A, luzonidial B, luzonoside C, luzonoside D, luzonoid A, luzonoid B, luzonoid C, luzonoid D, luzonoid E, luzonoid F, luzonoid G,	—	Leaves and twigs	Antitumor effects	[31]
*V. macrocephalum*	Methyl (2-*α*-L-rhamnopyranosyloxy)acetate, methyl (2R-3-*α*-L-rhamnopyranosyloxy)glycerate, methyl (3R-4-*α*-L-rhamnopyranosyloxy-3-hydroxy)butanoate, bridelionoside B (4), (6S,7E,9R)-roseoside, linarionoside A, 3,7,11-trimethyl-1,6-dodecadien-3,10,11-triol, (+)-8-hydroxylinalool, beta-sitosterol and daucosterol	Ethanolic extract	Branch	Insecticidal and antimicrobial activities	[61]
	Apigenin-7-*O*-[6-*O*-(5-methoxy-3-hydroxy-3-methyl-5-oxovaleryl)]-beta-D-glucopyranoside, kaempferol-3-*O*-(6^″^-*O*-acetyl)- *β*-D-glucopyranoside, kaempferol-3-*O*-(6^″^-*O*-crotonyl)- *β*-D-glucopyranoside, kaempferol 4′-*O*-*α*-L-rhamnopyranoside, (+)-naringenin-7-*O*-*β*-D-glucopyranoside, (–)-naringenin-7-*O*-*β*-D-glucopyranoside, afzelin, apigenin-7-*O*-*β*-D-glucopyranoside	Ethanolic extract	Branch	—	[84]
*V. melanocarpum*	(7R,8S)-Guaiacylglycerol4-*O*-*β*-D-(6-*O*-vanilloyl) glucopyranoside.	Ethanolic extract	Fruits	Intestinal alpha-glucosidase inhibitory activity	[85]
	(−)-(7R,7′R,8S,8′S)-Pinoresinol 4′-*O*-*β*-dglucopyranosyl-4-*O*-(6-*O*-vanilloyl)-*β*-d-glucopyranoside, (7′E,7S,8R)-7,9,9′-trihydroxy-3,3′,5′-trimethoxy-8-O-4′-neolignan-4-*O*-[6-*O*-(4-*O*-*β*-D-glucopyranosyl)vanilloyl]-*β*-d-glucopyranoside, pinoresinol 4,4′-*O*-*β*-dglucopyranoside, pinoresinol 4′-*O*-*β*-d-glucoside, syringaresinol 4′-*O*-*β*-d-glucopyranoside, pinoresinol 4-*O*-*β*-d-glucopyranosyl-(1→6)-*β*-d-glucopyranoside	Ethanolic extract	Stems	Inhibitory activity against alpha-glucosidase	[86]
	(7R,8S)-Syringylglycerol 8-O--D-allopyranoside, (7S,8S)-syringylglycerol 8-O--D-allopyranoside.	Ethanolic extract	Stems	Radical scavenging and glucosidase inhibitory activities	[87]
*V. odoratissimum*	Vibsane, vibsanol I, 15-hydroperoxyvibsanol A, 14-hydroperoxyvibsanol B, 15-O-methylvibsanin U, 5,6-dihydrovibsanin B, 14,18-O-diacetyl-15-O-methylvibsanin U, vibsanin K	—	Leaves	—	[88]
	Vibsane, vibsanin B, vibsanin F, neovibsanin B, neovibsanin	—	—	—	[89]
	Vibsanin I, vibsanin L, 14-hydroxyvibsanin F, 14R^∗^,15-epoxyvibsanin C, 14S^∗^,15-epoxyvibsanin C	—	Leaves	—	[90]
	Vibsanol C, vibsanol D, vibsanol E, vibsanol F, vibsanol G, vibsanol H, vibsanin X	—	Leaves and twigs	—	[91]
	Vibsanin A, vibsanin B, vibsanin C, vibsanin D, vibsanin E, vibsanin F, vibsanin I, vibsanin L, vibsanin M, aldovibsanin A, aldovibsanin B, aldovibsanin C, 7-epialdovibsanin A, 5-epivibsanin G, 18-O-methylvibsanin G, 14-hydroxyvibsanin F, (14R^∗^)-14,15-epoxyvibsanin C, (14S^∗^)-14,15-epoxyvibsanin C, vibsanol A, vibsanol B, 6*β*-hydroxy-3-oxolup-20(29)-ene-27,28-dioic acid, 6*α*-hydroxy-3-oxolup-20(29)-ene-27,28-dioic acid, quercetine.	—	Leaves and twigs	—	[31]
	Vibsanin C, vibsanin H, dehydrovibsanin G, vibsanol, 9-aldehydevibsanol, (+)-9′-O-senecioyllariciresinol, (8Z)-10-epi-vibsanin C, (+)-9′-O-isovaleryllariciresinol	—	Leaves and branch	—	[92]
	5-epi-Vibsanin G, 18-O-methylvibsanin G, vibsanin M, aldovibsanin C	—	Leaves and flowers	—	[93]
	Vibsanin B, vibsanin E, vibsanol A, vibsanol B, 6*β*-hydroxylup-20(29)-en-3-oxo-27,28-dioic acid, 6*α*-hydroxylup-20-(29)-en-3-oxo-27,28-dioic acid, 6*α*-hydroxylup-20(29)-en-3-oxo-28-oic acid	—	Leaves and flowers	—	[94]
	Benzaldehyde, exo-2-methylnorbornane, cis-linalool oxide (furanoid), linalool, nonanal, isophorone, 4-oxoisophorone, trans-linalool oxide (pyranoid), methyl salicylate, decanal, methyl nonanoate, eucarvone, 1-[2-(1-hydroxy-1-methylethyl)cyclopropyl]-ethanone, nonanoic acid, methyl geranate, methyl o-anisate, *α*-ionone, geranyl acetone, pentadecane, hexadecane, *β*-eudesmol, heptadecane, methyl eudesmate, octadecane, hexahydrofarnesyl acetone, phthalic acid, decyl isobutyl ester, methyl palmitate, methyl linoleate, methyl linolenate, heneicosane, docosane	—	Flowers	—	[95]
	*β*-Amyrin, *α*-amyrin, stigmasta-4-en-3-one, ergosta-4,6,8(14),22-tetraen-3-one, Olean-12-en-3-one, lupeol, 3-hydroxyolean-12-en-1-one, 3-acetoxyolean-12-en-28-ol, 3-acetoxyolean-12-en-28-oic acid, 3,28-dihydroxyolean-12-ene, 3,28-dihydroxyurs-12-ene, 28-hydroxyolean-12-en-3-one, trans-phytol, betulin	—	Roots	—	[96]
*V. opulus*	Ascorbic acid, total phenolics, total anthocyanin	—	Leaves	—	[97]
	Methyl pentanoate, 3Z -Hexen-1-ol, n-heptanal, 2-pentylfuran, phenyl acetaldehyde, linalool oxide, terpinolene, L-linalool, n-nonanal, 2E, 6Z-nonadienal, 4-terpineol, *α*-terpineol, methyl salicylate, myrtenol, n-decanal, trans-carveol, geraniol, 2E-decanal, 2E, 4Z–decadienal, 2E, 4E-decadienal, *α*-copaene, rans-*β*-damascenone, trans-*α*-ambrinol, *α*-amorphene, germacrene D, *β*-ionone, *γ*-cadinene, *Δ*-cadinene, trans-cadina-1, 4-diene, *α*-calacorene, ledol, tetradecanal, *α*-muurolol, *α*-cadinol, pentadecanal, manool, n-heneicosane, phytol, n-docosane, n-tricosane	—	Air-dried whole plants	—	[83]
	Chlorogenic acid	—	Fruits	—	[98]
	Gallic acid, procyanidin B1, (+)-catechin, procyanidin B2, (−)-epicatechin, neochlorogenic acid, chlorogenic acid, rutin, isorhamnetin, isorhamnetin 3-O-rutinoside, quercetin, anthocyanins, cyanidin-3-O-sambubioside, cyanidin-3-O-glucoside, cyanidin-3-O-rutinoside	—	Fruits		[99]
	Coumaroyl-quinic acid, chlorogenic acid dimer, procyanidin B2, catechin, procyanidin trimer epicatechin, proanthocyanidin dimer monoglycoside, quercetin-hexose + pentose, rutin, quercetin-hexose, quercetin-deoxyhexose	—	Fruits	—	[100]
	Quinic acid, catechin dimer, catechin, chlorogenic acid (3-O-caffeoylquinic acid), procyanidin C1, epicatechin, neochlorogenic acid (5-O-caffeoylquinic acid)	—	Fruits	—	[101]
	Ethyl alcohol, 1-propano, 2-butanone, acetic acid, ethyl acetate, isobutanol, 2-pentanone, 3-methyl-1-butano, 2-methyl-1-butanol, 1-pentanol, 2-hexanone, 2-hexanol, hexanal, 3-methyl-butanoic acid, 2-methyl-butanoic acid, 3-hexen-1-ol (Z), 1-hexanol, 2-heptanone, 2-heptanol, heptanal, 3-methyl-pentanoic acid, 1-heptanol, 1-octen-3-ol, 6-methyl-5-hepten-2-one, 2-octanone, ethyl hexanoate, 2-octano, octanal, hexyl acetate, a-terpinene, p-cymene, limonene, 1,8-cineole, trans-linalool oxide (furanoid), 2-nonanone, linalool L, nonanal, dill ether, *α*-terpineol, ethyl decanoate, *β*-caryophyllene (E)	—	Fruits	—	[101]
	Gallic acid, ascorbic acid, vitamin C	—	Fruits	—	[102]
	L-Malic acid, L-ascorbic acid, oxalic acid	—	Fruits	—	[103]
	Chlorogenic acid, (+)-catechin, (-)-epicatechin, cyanidin-3-glucoside, cyanidin-3-rutinoside and six different glucosides of quercetin.	—	Fruits	—	[104]
	Chlorogenic acid, oxalic acid, citric acid, tartaric acid, malic acid, quinic acid, succinic acid, fumaric acid, procyanidin B2, (−)-epicatechin, p-coumaric acid, isorhamnetin 3-O-rutinoside, isorhamnetin 3-O-glucoside, quercetin 3-O-glucoside.	—	Fruits, flowers, and bark	—	[34]
	*β*-Sitosterol, stigmasterol, colesterol, *α*-amyrin-urs-12-en-3-*β*-ol, *β*-amyrin-Olean-12-en-3-*β*-ol, lupeol, 3-keto-urs-12-ene, 3-keto-Olean-12-ene, A:D-neoolean-12,14-diene, A-neoolean-5,12-diene	—	Seeds	—	[105]
*V. orientale*	Chlorogenic acid.	Methanolic extract and aqueous	Fruit, leaf, and branch	Enzyme inhibitory and antioxidant effect	[106]
	Betulalbuside A, anatolioside E, betulalbuside B, anatolioside, anatolioside A, anatolioside B, anatolioside C, anatolioside D,	—	Leaves and twigs	Antitumor effects	[31]
*V. phlebotrichum*	Phlebotrichin, p-hydroquinone, arbutin,	—	Leaves and twigs	Antitumor effects	[31]
*V. plicatum*	Dideoxyplicatumoside A, erythro-syringylglycerol-*β*-*O*-4′-(+)-isoeucommin A 4^″^-*O*-*β*-D-glucopyranoside,	—	Leaves	—	[107]
	Plicatumoside A, (+)-neomedioresinol 4,4′-di-*O*-*β*-D-glucopyranoside, (+)-neomedioresinol 4,40-*O*-di-*β*-D-glucopyranoside	—	Leaves	—	[108]
	7-*O*-Tigloylsecologanol, 7-*O*-tigloylsecologanolic anolic acid, 3′-*O*-[(2S)-2-methylbutanoyl]henryoside, (4R)-*α*-terpineol *O*-*β*-D-glucopyranoside2), (7S,8R)-dihydrodehydrodiconiferyl alcohol 9-*O*-*β*-d-glucopyranoside2), (7R,8S)-dihydrodehydrodiconiferyl alcohol 9-*O*-*β*-d-glucopyranoside2), quercetin 3-*O*-robinobioside2), quercetin 3-*O*-rutinoside2), kaempferol 3-*O*-robinobioside2), kaempferol 3-orutinoside2).	Methanolic extract, chloroform, ethyl acetate, butanol, water	Leaves	—	[109]
*V. propinquum*	(3,4,2′,4′-tetrahydroxy-trans-chalcone), (3,4,2′,4′-tetrahydroxy-trans-chalcone-2′-*O*-*β*-D-glucoside), quercetin, (+)-dihydroquercetin, eriodictyol, taraxerol, *β*-sitosterol, stigmasterol, 3*β*,28-dihydroxy-12-ursene, ursolic acid, daucosterol, 4,2′,4′-trihydroxy-dihydrochalcone, 4,2′,4′-trihydroxy-dihydrochalcone-2′-*O*-*β*-D-glucoside.	—	Leaves and stems	Antioxidant activity	[110]
*V. prunifolium*	Scopoletin	—	Haw	Antispasmodics	[111]
	2′-Acetyldihydropenstemide, 2′-acetylpatrinoside, patrinoside, 2′-(E)-*p*-coumaroyldihydropenstemide	—	Leaves and twigs	Antitumor effects	[31]
*V. punctatum*	Phytosterols, triterpenoids, and phenolic compounds and their glycosides	—	Roots	—	[64]
*V. rhytidophyllum*	Ursolic acid, 7,10,2′-triacetylpatrinoside, 7-*p*-coumaroylpatrinoside, 10-acetylpatrinoside, catechin, arbutin, henryoside, salicin, viburnine	—	Leaves and twigs	Antitumor effects	[31]
*V. sargentii*	(-)-Epicatechin, 5,7,4-trihydroxy-flavonoid-8-C--d-glucopyranoside, 1-(4-hydroxy-3-methoxyphenyl)-2-[4-(3--l-rhamnopyranoxypropyl)-2-methoxyphenoxy]-1,3-propane-diol (erythro), 1-(4-hydroxy-3-methoxyphenyl)-2-[4-(3--l-rhamnopyranoxypropyl)-2-methoxyphenoxy]-1,3-propanediol (threo), (R)-4-hydroxylphenol *O*-(6-*O*-oleuropeoyl)--d-glucopyranoside, (R)-3-methoxy-4-hydroxylphenolO-(6-*O*-oleuropeoyl)--d-glucopyranoside, quercetin-3-*O*-rutinoside.	Ethanolic extract and aqueous	Fruits	Antioxidant activity	[112]
*V. suspensum*	Neovibsanin, vibsanins B, vibsanins F, vibsanin F, neovibsanin B.	—	—	—	[89]
	Neovibsanin F, gomojoside A, gomojoside B, gomojoside C, gomojoside D, gomojoside E, gomojoside F, gomojoside G, gomojoside H, gomojoside I, gomojoside J, gomojoside K, gomojoside L, gomojoside M, gomojoside N, gomojoside O, gomojoside P, gomojoside Q, 3-oxooleana-11,13(18)-dien-28-oic acid, 24-hydroxy-3-oxooleana-11,13(18)-dien-28-oic acid, 6*β*-hydroxy-3-oxooleana-11,13(18)-dien-28-oic acid, 2′,6′-*O*-diacetylscopolin.	—	Leaves and twigs	Antitumor effects	[31]
*V. tinus*	3-*O*-*β*-D-Galactopyranosyl-(1 → 2)-*O*-*β*-D-glucuronopyranosideoleanolic acid 28-*O*-*β*-D-glucopyranosyl ester, 3-*O*-(*β*-D-glucuronopyranosyl)oleanolic acid, 28-*O*-*β*-D-glucopyranosyl ester, oleanolic acid, viburtinoside A, viburtinoside B, viburtinoside I, viburtinoside II, viburtinoside III, viburtinoside IV, viburtinoside V, suspensolide F, suspensolide A, isoquercitroside, kaempferol 3-*O*-*β*-D-galactopyranoside, quercetine, nobiletin, rutin, afzelin, scopoletin 7-*O*-sophoroside, 2,6-Di-C-methylnicotinic acid 3,5-diethyl ester	—	Leaves and twigs	Antitumor effects	[31]
*V. urceolatum*	*α*-Amyrin palmitate, lupeol palmitate, *β*-amyrin acetate, ursolic acid, urceolatoside A, urceolatoside B, urceolatoside C, urceolatoside D, urceolide,	—	Leaves and twigs	Antitumor effects	[31]
*V. wrightii*	*α*-Amyrin palmitate, ursolic acid, astragalin, kaempferol 3-*O*-*β*-D-galactopyranoside, kaempferol 3-*O*-rutinoside, apigenin 7-O-*β*-D-glucoside, arbutin, *p*-hydroxypheny *β*-D-allopyranoside, 6-*O*-acetylarbutin, 4′-hydroxycinnamic acid, viburnolides A, viburnolides B, viburnolides C	—	Leaves and twigs	Antitumor effects	[31]
